# Incidence and Outcome of Unexpected Non-Cardiac Abnormality on Cardiac Magnetic Resonance Imaging: A Report from Northeastern Thailand

**DOI:** 10.3390/tomography7020018

**Published:** 2021-05-19

**Authors:** Narumol Chaosuwannakit, Pattarapong Makarawate

**Affiliations:** 1 Radiology Department, Faculty of Medicine, Khon Kaen University, Khon Kaen 40002, Thailand; 2 Cardiology Unit, Internal Medicine Department, Faculty of Medicine, Khon Kaen University, Khon Kaen 40002, Thailand; nchaosuw@gmail.com

**Keywords:** non-cardiac abnormality, cardiac MRI, incidental findings, mortality

## Abstract

*Objective:* To ascertain non-cardiac abnormality (NCA) incidence in patients undergoing clinical cardiovascular magnetic resonance imaging (CMR) and determine such patients’ clinical importance. *Methods:* Consecutive patients undertaking CMR study from January 2012 to June 2017 for various cardiovascular diseases were enrolled. To assess NCA’s therapeutic importance, all incidental findings that were not expected from the patient’s history were analyzed. A careful review of medical history determines the information on mortality. *Results:* Three hundred and eighty-two consecutive patients (mean age 58 ± 11 years) who underwent CMR for different clinical indications were enrolled in the present study. Potentially significant results have been identified as abnormalities that require further clinical or radiological follow-up or therapy. On CMR, 118 NCA (30.9%) were found. In 25 patients, potential clinically significant NCAs, such as aortic aneurysm (n = 3), aortic dissection (n = 2), pulmonary thromboembolism (n = 2), and malignancy (n = 18), were identified (6.54%). In terms of one-year mortality data, in a patient without NCA, we observed a significantly higher survival rate than those with NCA (*p* = 0.0085) and a higher mortality rate in a patient with clinically significant NCA than a patient with irrelevant NCA (*p* = 0.02). Survival, as assessed via Kaplan‒Meier analysis, disclosed significantly higher mortality in the patients with clinically significant NCA than patients with irrelevant NCA (HR = 11.20, CI = 4.71–26.60, *p* < 0.001). *Conclusions:* We concluded that it is vital for the CMR study to determine the relevance of NCA, especially in the cholangiocarcinoma endemic region such as northeastern Thailand. Eventually, to reorganize the patients according to appropriate management, clinical correlation and prognosis must be summarily established.

## 1. Introduction

Cardiovascular magnetic resonance imaging (CMR) has been established in clinical practice to diagnose and manage cardiovascular system diseases. It is a highly effective non-ionizing and non-invasive imaging technique [1]. Although the main components of a CMR study are acquired in specific imaging planes oriented along the axes of the heart, provisional axial and coronal images of the thorax and upper abdomen are also acquired to assist in study planning. It incorporates a large field of view on localizer sequences that offer coverage of the thorax and upper abdomen, which might disclose incidental non-cardiac abnormality (NCA) [2,3]. The NCA diagnosis is essential, and it has an impact on the next step of management, as well as the significance of incidental findings. The current study’s objective is to determine the incidence of NCA and the clinical outcome and mortality of clinically significant NCA that is unexpectedly detected on routine clinical CMR.

## 2. Materials and Methods

### 2.1. Patient Population

Three hundred and eighty-two patients who underwent clinical cardiovascular MRI (CMR) during January 2012–June 2017 were included in this retrospective cohort study. The current study was approved by Research Number HE 611,296 of the Ethics Committee of the Faculty of Medicine, Khon Kaen University, Khon Kaen, Thailand. Patients’ medical history with an incidental non-cardiac abnormality was reviewed to identify the next steps of the investigation, definitive diagnosis, treatment, and mortality over a one-year follow-up duration. The inclusion criteria were consecutive patients who underwent routine clinical CMR from various clinical indications; most of them are the evaluation of cardiomyopathy (34.6%), ischemic heart disease (30.9%), or congenital heart disease (29.1%) (Table 1). Exclusion criteria were incomplete medical information.

### 2.2. Cardiovascular Magnetic Resonance (CMR) Scanning Protocol

All cardiovascular sequence CMR exams were conducted according to the recommendation of standardized CMR guidelines and customized to each patient to address the specific clinical questions [4]. For patients who underwent more than one CMR examination, the present study included just the first scan. All CMR was conducted on a 1.5 T Siemens Avanto system (Siemens Medical Solutions, Erlangen, Germany), using a 16-element, phased-array surface coil, following standard electrocardiographic and respiratory gating protocol. To cover an anatomical range from the thyroid down to the upper abdomen for the anatomical localizer, half-Fourier acquisition single-shot turbo spin-echo (HASTE) and steady-state free precession (SSFP) sequences were used. These anatomical sequences were the most important for evaluating non-cardiac abnormality (NCA) as they had the most generous anatomical coverage among all CMR imaging sequences. For all anatomical imaging sequences, slice thickness was kept between 6 and 8 mm without a gap. For evaluating the right and left ventricular function, the standard protocol included multiplane T1-turbo spin-echo (TSE) black blood, cine steady-state free precession (SSFP) oriented to three-, two-, and four-chamber and short-axis views. T2-TSE black blood was used for the study of myocardial edema, and phase contrast was used to study pressure gradient and flow. Phase-sensitive inversion recovery (PSIR) sequences for the evaluation of myocardial scar and fibrosis using late gadolinium enhancement (LGE) were performed 10–15 min after intravenous administration of gadolinium (0.1 mmol/kg).

### 2.3. CMR Imaging Analysis and Follow-Up Protocol

An experienced radiologist evaluated each CMR examination retrospectively and identified the unexpected NCA. In the initial reports and clinical records, the interpreting radiologist was blinded. After the CMR exam, the median follow-up time was 10.6 months. The information included the search for further investigation, definitive clinically significant NCA diagnosis, and the review of clinical or histological findings following surgical or interventional procedures. The attributes of patients were analyzed and summarized. Cautious retrospective evaluation of the medical record was used to assess mortality data, resulting in 1-year survival data.

The data is stratified according to those with incidental findings, both irrelevant NCA and clinically significant NCA. The definition of clinically significant findings is the findings that further therapeutic or radiological follow-up or treatment is required [4,5,6]. To determine NCA’s clinical consequences and mortality rate, the follow-up results were analyzed by reviewing the hospital’s electronic medical reports database.

### 2.4. Statistical Analysis

The mean and standard deviation were used to express continuous data. A statistically significant result was described as one with a significance level of *p* < 0.05, and all identified *p*-values were two-sided. Means were compared using an unpaired t-test. When the data were not normally distributed, the Mann-Whitney rank sum was used to compare the means. Patients with clinically significant NCA and patients with irrelevant NCA had their hazard ratios, and Kaplan-Meier curves were analyzed. Hazard ratio and Kaplan-Meier curve analysis for patients with clinically significant NCA and patients with irrelevant NCA were also evaluated. Analyses were performed using SPSS version 19.0 (SPSS Inc., Chicago, IL, USA).

## 3. Results

In the current study, 382 consecutive patients (mean age 58 ± 11 years) undergoing CMR were recruited for diverse medical indications. Cardiomyopathy, ischemic heart disease, and congenital heart disease were the main CMR indications.

**Figure 1:**
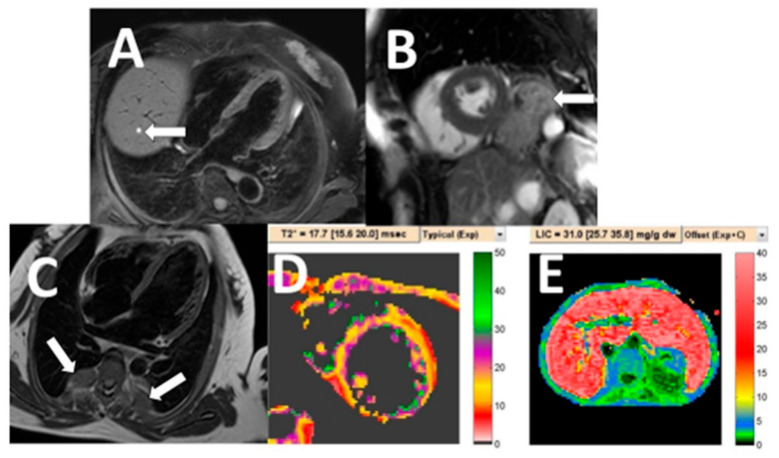
Examples of irrelevant non-cardiac abnormality on cardiac MRI. A cardiac MRI black blood image of a 52-year-old woman revealed a small, simple liver cyst (**A**: arrow). Cardiac MRI white blood image in short-axis plane detected hiatal hernia (**B**: arrow). Incidentally found extramedullary hematopoiesis (**C**: arrows) of thalassemia patient who underwent CMR for iron concentration quantification in the heart (**D**) and liver (**E**).

**Figure 2:**
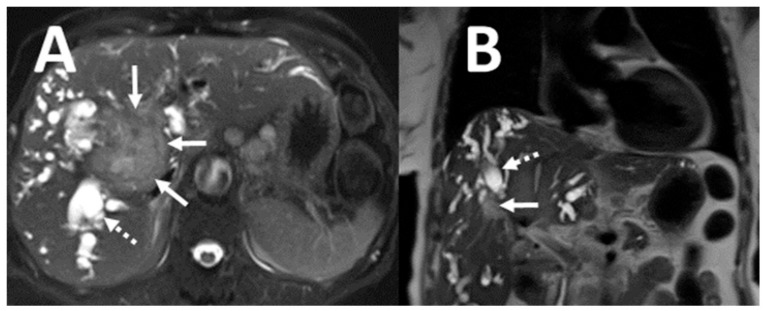
Example of clinically significant non-cardiac abnormality on CMR. Unexpectedly found hilar cholangiocarcinoma (**A**,**B**: solid arrows) with intrahepatic bile duct dilatation (**A**,**B**: dashed arrows).

**Figure 3:**
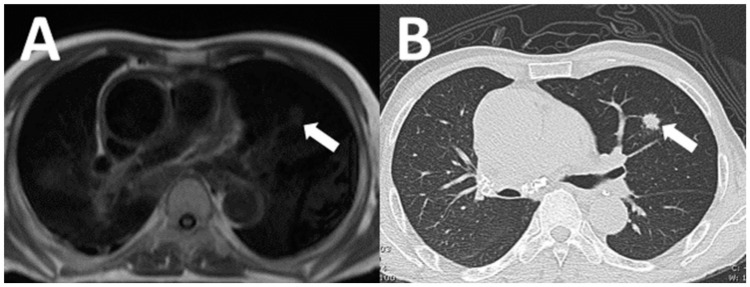
A 72-year-old man underwent CMR to evaluate myocardial viability. Incidentally detected lung nodule on axial black blood image cardiac MRI (**A**: arrow). Subsequent CT chest demonstrated speculated lung nodule (**B**: arrow) and was consequently diagnosed as primary lung cancer by CT-guided lung biopsy.

**Figure 4:**
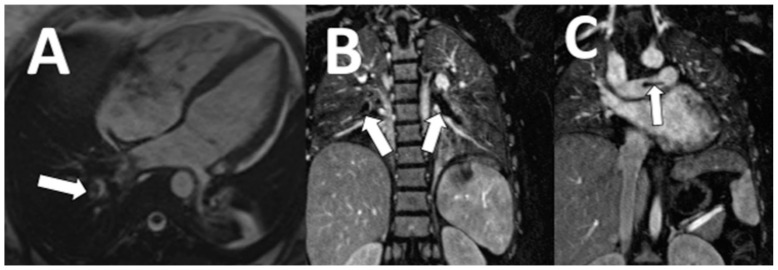
A 49-year-old woman underwent CMR for diagnosis of cardiomyopathy. Unexpectedly identified pulmonary thromboembolism in the right and left pulmonary arteries are demonstrated on white blood image (**A**: arrow) and coronal thoracic MRA (**B**,**C**: arrows).

Baseline characteristics of patients and cardiac MRI indications are presented in Table 1. There were no statistical differences between patients with NCA and those without NCA in age, sex, underlying disease, and CMR indications (Table 1). We found 118 (30.9%) non-cardiac abnormalities (NCA). The median follow-up time was 10.6 months (4–12 months). Most patients had a higher prevalence of irrelevant NCA, particularly renal cortical cysts, liver cysts, ascites, pleural effusion, or extramedullary hematopoiesis (Figure 1 and Table 2). In 25 patients (6.54%), clinically significant NCA was identified, such as cancer (n = 18) (Figure 2 and Figure 3), aortic aneurysm (n = 3), aortic dissection (n = 2), or pulmonary thromboembolism (n = 2) (Figure 4) (Table 2). Cholangiocarcinoma (n = 7) is the most unexpectedly diagnosed cancer on CMR in the present study ((Figure 2).

**Figure 5:**
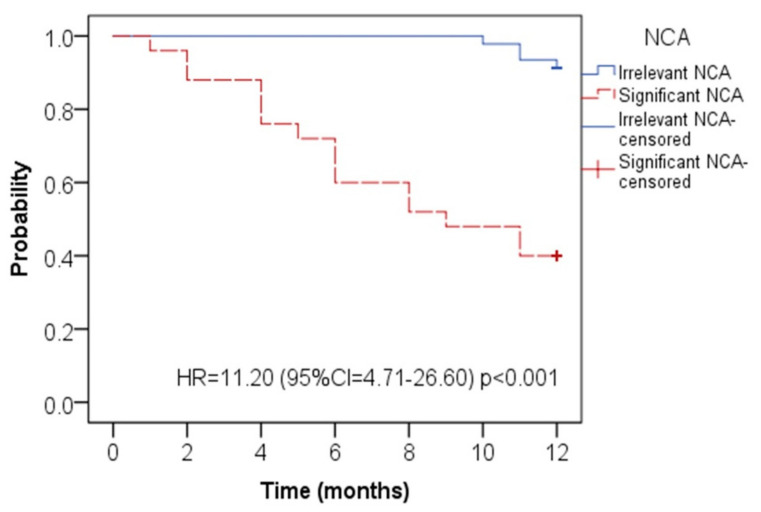
Kaplan-Meier curve for death events for the patients with clinically significant NCA (red line) and irrelevant NCA (blue line). (NCA: non-cardiac abnormality).

We detected a significantly higher survival rate in a patient without NCA relative to those with NCA (*p* = 0.0085) and a higher mortality rate in a patient with clinically significant NCA compared to a patient with irrelevant NCA (*p* = 0.02) in terms of one-year mortality data (Table 3). Survival, as assessed using Kaplan‒Meier analysis, disclosed significantly higher mortality in the patients with clinically significant NCA than patients with irrelevant NCA (HR = 11.20, CI = 4.71–26.60, *p* < 0.001) (Figure 5).

## 4. Discussion

The current study demonstrated 118 (30.9%) non-cardiac anomalies (NCA) detected on CMR that complied with previous studies that reported up to 26.4% of patients who underwent clinical CMR with incidental non-cardiovascular findings [7,8].

Renal cysts, liver cysts, ascites, and pleural effusion were the majority of irrelevant NCAs, and the majority of clinically significant NCAs were malignant tumors. While these results appear to be relatively high, they can be assessed by the population’s demographic nature as we image elderly patients with several comorbidities. In addition, we found seven patients with incidental cholangiocarcinomas (CCAs) incidentally diagnosed, and six of them died during the one-year follow-up period because our hospital was located in the cholangiocarcinoma endemic region [9]. A significant proportion of patients undergoing clinical CMR for ischemic heart disease evaluation had atherosclerosis risk factors, known as risk factors for a range of malignancies [10].

Hepatocellular carcinoma (HCC), which arises from hepatocytes, and cholangiocarcinoma, which arises from bile ducts, are two main forms of primary liver cancer with distinct histological features and origins (CCA). Hepatitis B and C infection have a closer correlation to HCC, the most common liver cancer. CCA is an unusual tumor in the rest of the world, but it is prevalent in Thailand, especially the northeastern part of Thailand. The carcinogenic liver fluke Opisthorchis viverrini, which is prevalent in this area, is implicated in epidemiological and laboratory evidence as a significant risk factor for CCA [11]. CCA is usually recognized as a fatal tumor with a poor prognosis, which includes Thai cases. CCA prognosis is determined by a range of factors, including tumor type, staging, free surgical margin, and distant metastasis. Thai patients with intraductal growth type CCA have a longer survival rate than CCA patients in other series, while the median survival time in diffuse-type CCA is just 65 days [12].

The importance of incidental non-cardiovascular findings was shown in the present study. We detected a significantly higher survival rate in patients without NCA than in patients with NCA (*p* = 0.0085) and a higher mortality rate in patients with clinically significant NCA (*p* = 0.02) than in patients with irrelevant NCA.

With an excellent diagnostic accuracy, CMR could be extremely useful in characterizing specific incidental findings, while additionally, imaging techniques are often required to define incidental findings precisely. CT scans, in particular, are the first method for identifying and determining the nature of incidental lung lesions. An ultrasound (US) or a dedicated CT or MRI could be used to further assess abdominal and breast lesions.

The current study has potential limitations, despite promising initial findings. First, we could only collect data on patients who returned to our health care system for consequent treatment due to retrospective study design. No result data was available on a group of patients who missed follow-up. Second, the present study’s sample size was relatively small, but this highlights our findings’ significance. Despite the limited sample size, there was substantial mortality associated with unexpected NCAs. Third, our findings represent a single-center experience, with restricted generalizability of the results reported. Finally, our definition of significant and non-significant incidental findings was broad; however, the stratification we used was compatible with previous studies [6,7,8,13,14,15,16]. Eventually, the best inclusion indicator in the significant clinical group was the follow-up and clinical correlation.

## 5. Conclusions

A significant portion of the neck, thorax, and upper abdomen is imaged throughout routine clinical CMR. Non-cardiac abnormalities (NCAs) are common, and an essential proportion of these findings are clinically relevant. We propose that it is the ethical and technical duty of physicians reporting CMR to be adequately competent in interpreting extracardiac anomalies or to consult with trained colleagues. It is valuable for the patient to interpret NCAs correctly. Like many others, we contemplate that the best way is to examine all possible data in each CMR study and not just a small field of view series and report all NCA results followed by their clinical significance.

## Figures and Tables

**Table 1:** Baseline patient characteristics and cardiac MRI indications.

Variable	Patients with NCA (n = 118)	Patients without NCA (n = 264)	*p*-Value
**Age; years (mean ± SD)**	55.6 ± 11.2	57.1 ± 14.3	0.34
Female	56 (47.5)	134 (50.8)	0.55
Obesity *	11 (9.3)	28 (10.6)	0.77
Diabetes	30 (25.4)	65 (24.6)	0.87
Hypertension	58 (23.7)	78 (29.5)	0.24
Dyslipidemia	42 (35.6)	73 (27.7)	0.12
Smoking history	10 (8.5)	24 (9.1)	0.85
Previous cardiac surgery	44 (37.3)	101((38.3)	0.85
**Cardiac MRI indications**			
Cardiomyopathy	40 (33.9)	92 (34.8)	0.86
Ischemic heart disease	38 (32.2)	80 (30.3)	0.71
Congenital heart disease	33 (27.9)	78 (29.5)	0.75
Cardiac mass	4 (3.4)	8 (3.0)	0.83
Pericardial disease	3 (2.6)	7 (2.4)	0.91

Data presented as n (%) unless indicated otherwise; NCA: non-cardiac abnormality, * body mass index > 30 kg/m^2^.

**Table 2:** Incidental non-cardiac abnormality (NCA) on cardiac MRI.

Non-Cardiac Abnormality (NCA)	n (%)
**Clinically significant NCA (n = 25)**	
Malignancy	18 (72)
Cholangiocarcinoma	7 (28)
Lung cancer	4 (16)
Hepatocellular carcinoma	3 (12)
Breast cancer	1 (4)
Renal cell carcinoma	2 (8)
Bone metastasis	1 (4)
Pulmonary thromboembolism	2 (8)
Aortic dissection	2 (8)
Aortic aneurysm	3 (12)
**Irrelevant NCA (n = 93)**	
Simple renal cortical cyst	36 (38.7)
Simple liver cyst	11 (11.7)
Hepatic hemangioma	2 (2.2)
Ascites	10 (10.7)
Splenic cyst	1 (1.1)
Splenomegaly	4 (4.3)
Esophageal dilatation	6 (6.5)
Pleural effusion	8 (8.6)
Pleural plaque	2 (2.2)
Hiatal hernia	3 (3.2)
Thyroid goiter	4 (4.3)
Extramedullary hematopoiesis	5 (5.4)
Thoracic spine hemangioma	1 (1.1)

**Table 3:** Comparison of one-year mortality between clinically significant NCA and irrelevant NCA groups and comparison of one-year mortality between patients without NCA and patients with NCA.

One-Year Mortality	Clinically Significant NCA	Irrelevant NCA	*p*-Value
Death	15 (60)	8 (9.4)	0.02 *
Survived	10 (40)	85 (90.6)	0.0017 *
**One-Year Mortality**	**Patient without NCA**	**Patient with NCA**	***p*-Value**
Death	24 (9.1)	23 (19.5)	0.31
Survived	240 (90.9)	95 (80.5)	0.0085 *

NCA: non-cardiac abnormality; data presented as n (%) unless indicated otherwise; * statistically significant at *p*-value < 0.05.

## Data Availability

Data sharing is not applicable to this article.
